# The origin and evolution of sex chromosomes, revealed by sequencing of the *Silene latifolia* female genome

**DOI:** 10.1016/j.cub.2023.05.046

**Published:** 2023-06-07

**Authors:** Jingjing Yue, Marc Krasovec, Yusuke Kazama, Xingtan Zhang, Wangyang Xie, Shencheng Zhang, Xiuming Xu, Baolin Kan, Ray Ming, Dmitry A. Filatov

**Affiliations:** 1 Centre for Genomics and Biotechnology, Fujian Provincial Key Laboratory of Haixia Applied Plant Systems Biology, Key Laboratory of Genetics, Breeding and Multiple Utilization of Crops, Ministry of Education, Fujian Agriculture and Forestry University, Fuzhou, 350002, China; 2 Department of Biology, University of Oxford, Oxford, OX1 3RB, United Kingdom; 3 Sorbonne Université, CNRS, UMR 7232 Biologie Intégrative des Organismes Marins (BIOM), Observatoire Océanologique, 66650 Banyuls-sur-Mer, France; 4 Department of Bioscience and Biotechnology, Fukui Prefectural University, 4-1-1 Kenjojima, Matsuoka, Eiheiji-cho, Fukui 910-1195, Japan; 5 Shenzhen Branch, Guangdong Laboratory for Lingnan Modern Agriculture, Genome Analysis Laboratory of the Ministry of Agriculture, Agricultural Genomics Institute at Shenzhen, Chinese Academy of Agricultural Sciences, Shenzhen, 518100, China; 6 Key Laboratory of the Ministry of Education for Coastal and Wetland Ecosystems, College of the Environment and Ecology, Xiamen University, Xiamen, 361100, China

**Keywords:** sex chromosome evolution, recombination suppression, genome sequence, *Silene latifolia*

## Abstract

White campion (*Silene latifolia*, Caryophyllaceae) was the first vascular plant where sex chromosomes were discovered. This species is a classic model for studies on plant sex chromosomes due to presence of large clearly distinguishable X and Y chromosomes that originated *de novo* about 11 million years ago (MYA), but lack of genomic resources for this relatively large genome (~2.8 Gb) remains a significant hurdle. Here we report *S. latifolia* female genome assembly integrated with sex-specific genetic maps of this species, focusing on sex chromosomes and their evolution. The analysis reveals highly heterogeneous recombination landscape with strong reduction in recombination rate in the central parts of all chromosomes. Recombination on the X chromosome in female meiosis primarily occurs at the very ends and over 85% of the X-chromosome length is located in a massive (~330 Mb) gene-poor rarely recombining pericentromeric region (Xpr). The results indicate that the non-recombining region on the Y chromosome (NRY) initially evolved in a relatively small (~15Mb) actively recombining region at the end of the q-arm, possibly as a result of inversion on the nascent X chromosome. The NRY expanded about 6 MYA via linkage between the Xpr and the sex-determining region, which may have been caused by expanding pericentromeric recombination suppression on the X chromosome. These findings shed light on the origin of sex chromosomes in *S. latifolia* and yield genomic resources to assist ongoing and future investigations on sex chromosome evolution.

## Introduction

Sex chromosomes are a peculiar part of the genome that evolved independently many times in different groups of organisms ^1,2^. In vascular plants they were first discovered exactly a century ago by Kathleen Blackburn ^3^ who reported that *Silene latifolia* (then known as *Lychnis alba*) has “an XY pair of chromosomes in the male with a corresponding XX in the female”. Despite independent origins of sex chromosomes in different organisms, their properties are quite similar, indicating the generality of evolutionary forces and processes driving their evolution. Non-recombining sex-specific Y(or W)-chromosomes are typically genetically degenerate, while X(or Z)-chromosomes that actively recombine in the homogametic sex, contain hundreds to thousands of functional genes ^4^. The contrasting properties of the Y(or W)- and X(or Z)-chromosomes are striking, given they typically evolve from a pair of autosomes ^5^ and initially have the same gene composition ^6^. This represents a good illustration of how the differences in recombination landscape can drive evolution of major changes in chromosome structure, density of genes and repetitive DNA ^7–10^.

Evolution of a non-recombining sex-specific region is a key step in sex chromosome evolution, yet how and why recombination cessation on nascent sex chromosomes evolves remains poorly understood ^11–15^. Species with very small sex-determining regions (e.g. in fish ^16,17^ and plants ^18–22^) demonstrate that recombination suppression around the sex-determining gene(s) is not obligatory, raising questions why large non-recombining regions repeatedly evolved and expanded in size on sex chromosomes of many species independently ^11,14,15,23–27^. Species where large non-recombining regions around sex-determining genes evolved recently, such as *S. latifolia* ^28^ that is the focus of this study, allow us to study what causes evolution of recombination suppression on sex chromosomes ^15^.

Studies in *S. latifolia* have contributed significantly to our understanding of sex chromosome evolution ^29–31^. Early work in this species ^32^ has inspired the development of ideas about how separate sexes and sex chromosomes originate ^33^. More recent work brought important clues on Y chromosome degeneration in plants ^28,34–36^, sex-biased expression ^37^, dosage compensation ^36,38–40^ and specific sex linked gene evolution ^41–45^. The *S. latifolia* sex-chromosomes evolved *de novo* during or after the transition to separate sexes about 11 million years ago ^28^, that is relatively recent compared to mammals ^46^ or birds ^47^, where the sex chromosomes are at least 10 times older. The *de novo* evolution of X and Y chromosomes in *S. latifolia* offers a rare opportunity to study the origination of sex chromosomes once a species evolves separate sexes ^5^. Relatively recent origin of sex chromosomes in this species enables the analysis of evolutionary dynamics of sex chromosomes at the early stages of their evolution.

Fragmented assemblies of the *S. latifolia* female ^36^ and male ^28^ genomes remain a significant limitation in the work with this species. This fragmentation does not allow one to establish the order and location of genomic contigs and the locations of the recombining and non-recombining regions on the sex chromosomes and autosomes. Relatively large size of the genome (haploid ~2.8 Gb ^48,49^) and abundance of repetitive sequences has, so far, precluded the construction of better genome assemblies. In this study, we took advantage of multiple long read sequencing datasets to generate a new female assembly close to chromosome scale and integrate it with genetic maps. This revealed highly uneven recombination landscape throughout *S. latifolia* genome, with peripheral recombination in all chromosomes. Extensive pericentromeric recombination suppression (PRS) in females is particularly pronounced on the X chromosome, where it has likely contributed to evolution of sex chromosomes by facilitating or even causing the expansion of the NRY. Furthermore, the PRS on the X chromosome significantly affects the patterns of genetic diversity and divergence in the sex-linked genes that have previously been attributed to Y chromosome degeneration.

## Results

### Genome sequencing, assembly and annotation

**Figure 1:**
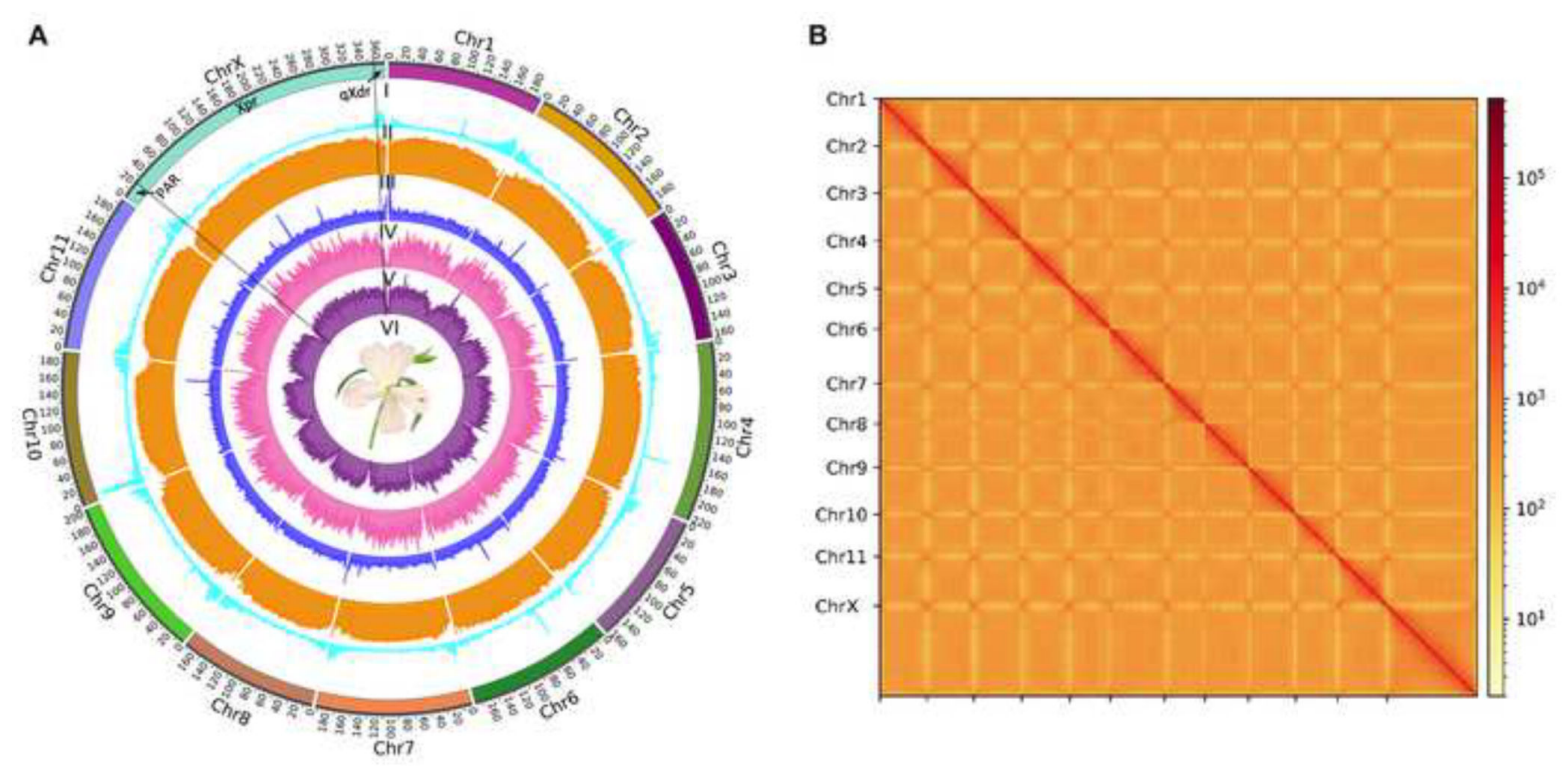
The genome assembly of *S. latifolia* female genome. A) Rings indicate twelve chromosomes (I), gene density (II), TE density (III), GC density (IV), copia LTR retrotransposons density (V), gypsy LTR retrotransposons density (VI). The dotted lines show the boundaries between PAR, Xpr and qXdr on the X chromosome. B) Hi-C chromatin interactions at 1 Mb resolution for twelve chromosomes. See also Tables S1-S9.

We used PacBio HiFi sequencing (104.9 Gb, 40× sequence coverage; Table S1) to create a draft assembly of the *S. latifolia* female genome. The initial assembly was 2.64 Gb long (contig N50=23.1 Mb; Table S2) with 98.0% genome completeness as assessed by BUSCO (Table S3). This assembly was then integrated with Hi-C data using ALLHiC pipeline ^50^. This yielded 12 pseudo-chromosomes with a total size of 2.45 Gb and an anchor rate of 93.0% (Figure 1A; Table S2). The number of assembled pseudo-chromosomes corresponds to the 12 chromosomes in the *S. latifolia* genome. The accuracy of Hi-C based pseudo-chromosome construction was evaluated by chromatin contact matrix, which showed a well-organized interaction contact pattern along the diagonals within each pseudo-chromosome (Figure 1B, Table S4). The quality of the female *S. latifolia* assembly was further verified by RNA-seq and genomic short Illumina reads from ^28,36^. The results showed that 100% of transcripts were aligned with 99.9% of single base accuracy for the genome (Table S5) and 99.1% of the genomic Illumina reads mapped to the assembly, covering 94.0% of the genome assembly (Table S6). All these results indicated the high quality of the assembled *S. latifolia* female genome.

**Figure 2:**
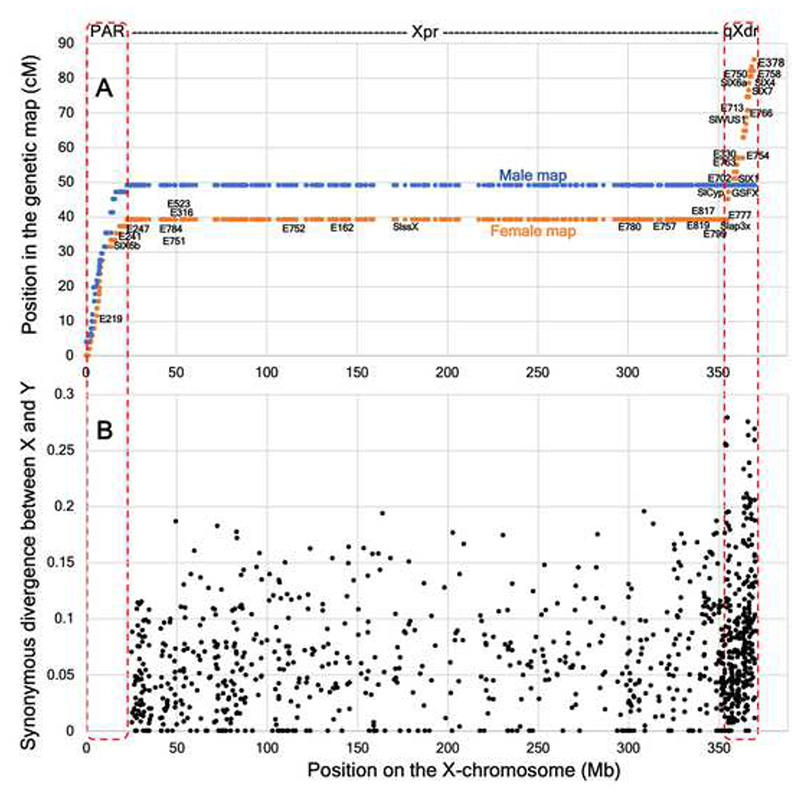
Genetic versus physical position for the X chromosome (A) and synonymous divergence between X- and Y-linked gametologs in the Xpr and qXdr (B). The names of genes on panel A show the locations of sex-linked genes genetically mapped in the previous studies ^35,43,45^. The genetic map of the PAR in panel A is longer in the male map due to obligatory sex chromosome paring in the PAR in male meiosis. See also Figure S1.

The assembled genome was integrated with the previously published RNA-seq-based sex-specific maps ^51^, which revealed a good correspondence between the genome sequence and the maps (Figures 2A and S1). In particular, 4745 and 4598 markers of the female- and male-specific maps ^51^, respectively, were located in the genome, of which 812 and 809 markers were X-linked. This analysis revealed that X-linked markers mapped to the scaffold “Chr12”, indicating its correspondence to the X chromosome. Furthermore, the sex-linked genes *SlX1, SlX3, SlX4, SlCyp* and *SlssX* previously located on the X chromosome with fluorescent *in situ* hybridisation ^52^, are all located on the scaffold “Chr12” (Figure 2A), confirming that this scaffold represents the sequence of the X chromosome.

We annotated 37,796 protein-coding genes in the *S. latifolia* female genome with 96.9% annotation completeness assessed by BUSCO (Tables 1 and S7). The average gene density on the X chromosome was significantly lower than that on the autosomes, except Chr4 and Chr6 (Table 1). We annotated 93 microRNAs (miRNAs) in *S. latifolia* female genome (Table S2). Annotation of repetitive sequences revealed that they represent 82.8% (2.18 Gb) of the genome assembly (Table S8). LTR retrotransposons (LTRs) were the most abundant transposable elements (TEs) representing 71.72% of the *S. latifolia* female genome (Figure1A, Table S8). Within the LTRs, Ty3/gypsy elements comprise the most abundant group, composing 35.9% of the genome, followed by Ty1/copia (12.8%) (Table S8). TEs were significantly more abundant on the X chromosome compared to autosomes, except Chr4 and Chr6 (Table 1), comprising 87.1% of the X chromosome, with 13.3% represented by Ty1/copia elements, and 37.3% by Ty3/gypsy elements (Figure 1A). The TEs comprise significantly higher proportion of the X chromosome compared to autosomes, except Chr4 and Chr6 (Table 1).

### Recombination suppression on the X chromosome

The comparison of the physical and genetic distances along the genomic scaffolds revealed reduced recombination in the central part of all chromosomes, indicating considerable pericentromeric recombination suppression in both sexes (Figure S1). In particular, the female meiosis recombination in the distal regions of the X chromosome is ~2 cM/Mb on average (Figure 2A), while the central region, comprising at least 85% of the X chromosome length, is rarely recombining (~0 cM/Mb). This is consistent with the view that species with large chromosomes tend to have highly peripheral recombination, with large pericentromeric regions lacking recombination ^53,54^.

One of the distal actively recombining regions in the X chromosome scaffold contains the genes previously identified ^36,51^ to be located in the pseudoautosomal region (PAR). As *S. latifolia* PAR is located in the p-arm of the X chromosome ^55–57^, we will refer to this region as the p-arm X distal region (pXdr or PAR; the left side on Figure 2), while the actively recombining region on the other side of the X chromosome will be referred to as q-arm X distal region (qXdr; the right side on Figure 2). The rarely recombining region between the pXdr and qXdr will be referred to as the X pericentromeric region (Xpr).

The PAR boundary (as defined in ^58^) is located close to the boundary between pXdr and Xpr. According to the previously published sex-specific genetic map ^51^, female recombination rate at the X-linked genes adjacent to the PAR boundary (the ‘right’ and ‘mid’ regions in ^58^) is zero, suggesting that they belong to the rarely recombining Xpr region. On the other hand, the pseudoautosomal genes adjacent to the PAR boundary (the ‘left’ region in ^58^) appear to recombine more actively, suggesting that they are located in the pXdr. Thus, it is likely that the PAR boundary coincides with the pXdr/Xpr boundary, though genetic mapping in several independent crosses is needed to confirm this result.

The genes previously used to define ‘evolutionary strata’ on *S. latifolia* sex chromosomes ^36,42,43,51,58–60^ – the regions where cessation of X:Y recombination occurred first (stratum 1: SlX4, SlX7, E766, E713, E758, E750, E378 ^35,42,43,60^) and then more recently (stratum2: SlssX, Slap3X, E799, E817, E819 ^35,43,61^) are located in the qXdr and the Xpr, respectively (Figure 2A). This indicates that initial cessation of recombination between the proto-X and proto-Y chromosomes around 11 MYA^28^ occurred in an actively recombining qXdr that is relatively small (~15 Mb long), while expansion of the NRY about 6 MYA involved inclusion of the massive (~330Mb) Xpr into the non-recombining male-specific region. Higher X:Y synonymous divergence in qXdr compared to Xpr (Figure 2B and Table 2), reported in the next section, is consistent with this conclusion.

The actively recombining regions at the ends of the X chromosome are gene-rich, while the Xpr is gene poor (Figure 1A, Table S9), with the average gene density of 17.8 and 6.6 genes per megabase, respectively. The distribution of TEs showed the opposite pattern, with TEs significantly more abundant in Xpr compared to the ends of the X chromosome (Figure 1A, Table S9). Similar TE depletion and gene enrichment is evident at the ends of most other chromosomes (Figure 1A).

### Substitution rates in X- and Y-linked genes

To reconstruct Y-linked gemetologs for the X-linked genes we employed previously described approach, which uses male-specific Y-linked SNPs to identify the sequence reads corresponding to the Y chromosome and assemble Y-consensus from these reads ^28,34,36^. The accuracy of the Y-reconstruction was confirmed in comparisons with the sequences of Y-linked genes previously obtained by manual Sanger sequencing (e.g. SlY1, SlY4, DD44Y, SlssY ^61–64^). The resulting Y-linked genes were aligned with X-linked gametologs as well as with their homologs (identified by blast) from a non-dioecious outgroup *Silene uniflora* ^65^.

Average synonymous divergence between the X- and Y-linked gametologs (d*S* X:Y) was 6.3% (±0.18%) and 9.1% (±0.42%) in the Xpr and the qXdr, respectively (Table 2 and Figure 2B). Consistent with relaxation of selection on the Y chromosome, the non-synonymous to synonymous substitution rate ratios (d*N*/d*S*) for the Y-linked genes were significantly higher compared to their X-linked gametologs in both Xpr and qXdr regions (0.591 vs 0.315 and 0.408 vs 0.267, respectively; Table 2).

The analysis of divergence between the genes in *S. latifolia* female genome and their homologs in outgroup *S. uniflora* (Table 2) revealed that d*N*/d*S* ratios of the X-linked genes in the qXdr are not significantly different from those in the pseudoautosomal genes (0.233±0.0186 and 0.221±0.0170, respectively; t-test, NS), while the genes in the Xpr have significantly higher *dN*/*dS* ratios (0.285±0.0123; t-test, *P*<0.001). This is consistent with reduced efficacy of selection in the rarely recombining Xpr on the X chromosome compared to the recombining PAR and qXdr that actively recombines in females.

### Gene expression

**Figure 3:**
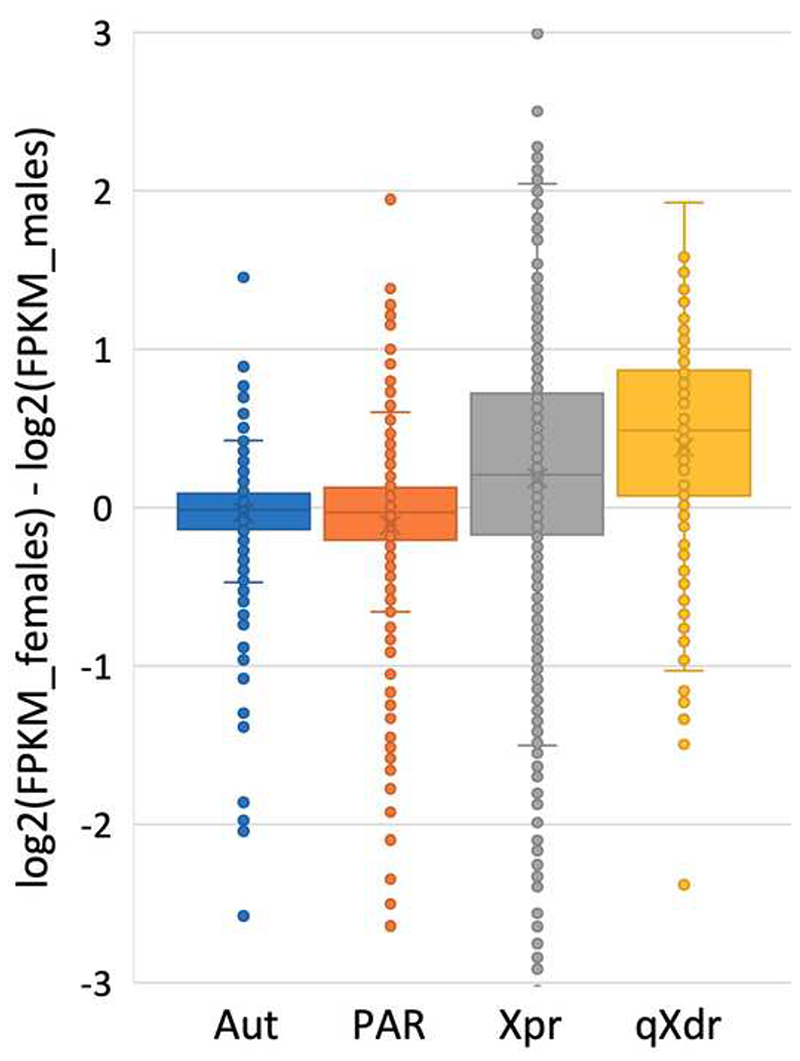
Female-biased expression of the genes in the qXdr and Xpr regions on the sex chromosomes. All comparisons between the groups, except the Aut:PAR comparison, are significant (t-tests, *P*<0.001).

Y-linked copies were significantly less actively expressed compared to their X-linked gametologs (paired t-test, *P*<0.0.001), reflecting degeneration of Y-linked genes at the level of gene expression. Overall (X+Y or X+X) expression is female-biased, while this is not the case in the PAR or autosomal genes (Figure 3). The presence of the female bias indicates that the dosage compensation system (if any is present in *S. latifolia* ^34,36,38,39,66^) is only partial and it does not adequately compensate for reduced expression of Y-linked genes. However, it is possible that the genes with reduced X+Y (compared to X+X) are not dosage sensitive and thus do not need to be compensated. Female bias is significantly stronger in the qXdr compared to the Xpr (t-test, *P*<0.001; Figure 3), indicating that the extent of Y-degeneration in gene expression is stronger for the Y-linked gametologs of qXdr than Xpr genes, possibly because the qXdr is the oldest part of the sex chromosome. Alternatively, weaker female bias in the Xpr compared to the qXdr is due to differences in female recombination rates in these regions. As Xpr is rarely recombining, the difference in recombination rate (and hence, efficacy of purifying selection keeping genes functional) between the X- and Y-linked genes is smaller compared to that for genes in qXdr that is actively recombining in females.

## Discussion

Lack of reference genome sequence for *S. latifolia* significantly limited the previous work devoted to evolution of sex chromosomes in this interesting system. Here we reported the assembly of the female genome and its integration with genetic maps ^36,51^, which shed light on structure and evolution of sex chromosomes in this species. It revealed that recombination in the *S. latifolia* genome primarily occurs in relatively small regions at the ends of the chromosomes (Figure S1), which is particularly pronounced on the X chromosome (Figure 2A) and may have played a role in sex chromosome evolution, as discussed below. Suppression of recombination in the region around the sex-determining gene(s) is a key step in NRY formation and sex chromosome evolution. Our results help to understand how such recombination suppression evolves, stressing the importance of the pre-existing recombination landscape on nascent sex chromosomes. They also illustrate that the real sequence of events leading to sex chromosome evolution may deviate significantly from the classic ‘evolutionary strata’ scenario ^67^ and the models of NRY expansion discussed in the literature ^14,24,26,27,68^.

### Evolution of recombination suppression on sex chromosomes

**Figure 4:**
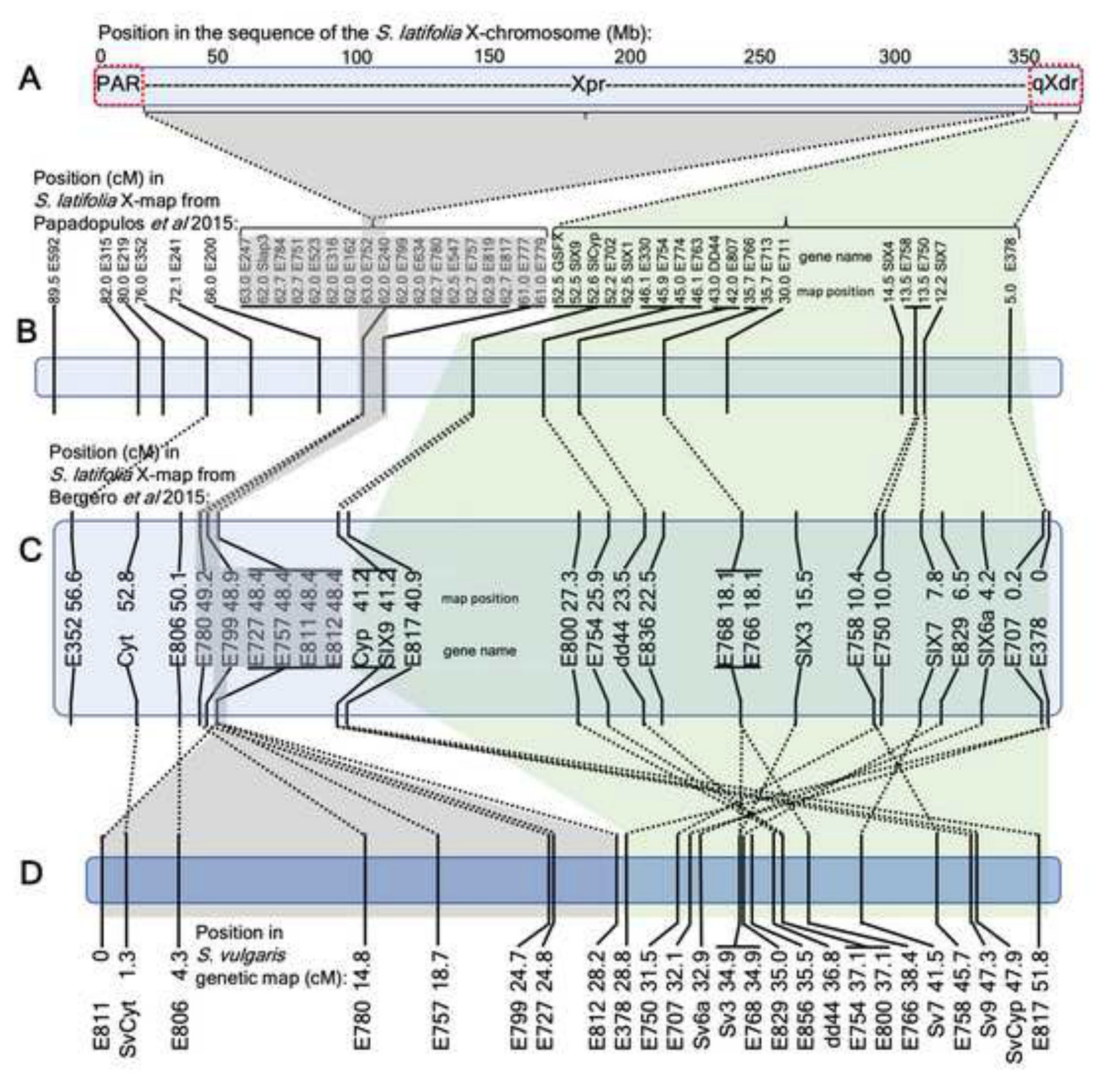
The comparison of the physical (A) and genetic (B-D) maps of the X chromosome in *S. latifolia* (A-C) and its homologous chromosome in non-dioecious *S. vulgaris* (D). Panel B shows the genetic positions from ^36^ for a subset of X-linked genes from this study that correspond to genes previously mapped in ^35,43^. Panels C and D show *S. latifolia* and *S. vulgaris* genetic maps from ^35^. The locations of the same gene in different maps are linked with dotted lines. Green and grey shading show the genes and regions corresponding to the qXdr and Xpr, respectively. Note that qXdr is long (i.e. actively recombining) in the genetic maps of both *S. latifolia* and *S. vulgaris*, while Xpr is long only in *S. vulgaris* and the genetic distance in this physically large region is nearly zero in *S. latifolia*, indicating evolution of suppressed recombination on the X chromosome in this region.

The size of the qXdr is only about 15 Mb, indicating that recombination suppression between the sex chromosomes in *S. latifolia* males has initially evolved in a relatively small region, not dissimilar to other dioecious plants, such as papaya ^18^, persimmon ^19^, kiwifruit ^20^, asparagus ^21^, ginkgo ^22^ etc. However, unlike papaya ^69^, kiwifruit ^70^ and *Rumex* ^71^ where the NRY evolved in a rarely recombining pericentromeric region, initial recombination suppression in *S. latifolia* has evolved in the actively recombining region (qXdr), where the GSFX – the X-linked gametolog of the putative sex-determining gene GSFY ^72^, is located (Figure 2A). This is consistent with the comparison of genetic maps in *S. latifolia* and *Silene vulgaris* that revealed the presence of active recombination in this region in both species (green shading in Figure 4). It is interesting that the order of genes in this region is inverted between the two species (Figure 4C-D), suggesting that the initial recombination suppression on the proto-Y chromosome in this region was caused by an inversion on the proto-X chromosome. However, without the data from other *Silene* species it is difficult to test whether this inversion arose in *S. latifolia* or *S. vulgaris* lineages.

Partial genetic maps are available for *Silene otites* and *Silene pseudotites* ^73^ that independently evolved separate sexes and homomorphic ZW and XY sex chromosomes corresponding to *S. latifolia* linkage groups LG3 and LG6, respectively. It is interesting that in both cases the sex-determining region corresponds to the central rarely recombining parts of homologous *S. latifolia* autosomes, which suggests that pre-existing pericentromeric recombination suppression facilitated evolution of sex chromosomes in *S. otites* and *S. pseudotites*, as it was also reported for *Rumex* ^71^. Unfortunately, the partial maps available for these *Silene* species focus on LG1, LG3 and LG6, which does not allow us to test whether the inversion in the *S. latifolia* stratum 1 of the X chromosome (Figure 4 C,D) occurred in *S. latifolia* lineage.

If confirmed that recombination between *S. latifolia* proto-X and Y chromosomes is suppressed by the X-linked inversion, this would contrast with the common assumption in the models that such inversions are Y- rather than X-linked ^24,26,68^. Many of the models developed with Y-linked inversions in mind do not work for X-linked inversions. For example, the deleterious mutations sheltering model ^24^ requires the inversion to be linked to a permanently heterozygous locus, such as the Y chromosome. However, some of the existing models may still work for X-linked inversions. For example, the crux of the recently proposed early emergence of dosage compensation model ^26^ is that evolving expression modifiers prevent reversion to X:Y recombination in the region where X:Y recombination stopped due to fixation of an inversion. While that model was developed with Y-linked inversions in mind, it may still work if X:Y recombination is suppressed by an X-linked inversion.

The comparison of the genetic map lengths in the Xpr (Figure 4B,C) with the homologous region in *S. vulgaris* (Figure 4D) reveals that recombination suppression is specific to the *S. latifolia* Xpr and thus may have evolved during the evolution of sex chromosomes in this species. It is interesting to speculate that the expansion of *S. latifolia* NRY to include Xpr may have been driven or facilitated by the evolution of pericentromeric recombination suppression in the central region of the X chromosome. For example, the NRY expansion could have occurred when the pericentromeric recombination suppression on the X chromosome became so extensive that it reached the sex-determining region. Comparative analysis of genetic and physical distances in a few other *Silene* species will be necessary to test this conjecture.

### Tip-biased distribution of recombination on *S. latifolia* chromosomes

Recombination suppression in central regions of the chromosomes is not universal in plants, with a lot of variation across species ^54^, but species with large chromosomes (>100 Mb) tend to have peripheral distribution of recombination ^53,54^. The concentration of crossovers at the ends of the chromosomes may be explained by mechanistic and/or adaptation-related causes. For example, the mechanistic ‘telomere-initiation’ model postulates that crossovers tend to occur at the ends of chromosomes because the recombination machinery starts at the telomeres and proceeds inward ^53,74^. Alternatively, distal distribution of recombination may be selected for to ensure sufficient recombination in gene-dense regions at the ends of large chromosomes ^54^. Regardless of whether the cause is mechanistic or adaptation-driven, the peripheral distribution of recombination may be fuelled by the tendency of ‘junk DNA’, such as transposable elements, to accumulate in gene-poor rarely recombining central chromosome regions ^7^ that hence expand over time, limiting the actively recombining gene-rich regions to the ends of large chromosomes. This ‘expanding junkyard’ model helps to explain why large chromosomes tend to have peripheral recombination, while on the smaller chromosomes this tendency is weaker, if present at all ^54^.

Given the association between the extent of peripheral recombination and the chromosome size ^54^, it is interesting to speculate that the expansion of *S. latifolia* genome may have contributed to evolution of recombination suppression between the X and Y chromosomes. The size of the *S. latifolia* genome (~2.8 Gb) is nearly three-fold larger compared to non-dioecious outgroups such as *S. vulgaris* (1.13 Gb ^48^) or *S. conica* (0.9 Gb ^49^). Many species in genus *Dianthus* that is closely related to *Silene*, have even smaller genomes (e.g. *Dianthus deltoides* genome ~0.45 Gb ^75^), making it likely that the large size of *S. latifolia* genome is a derived state. As the chromosome number in diploid *Silene* is mostly conserved (N=12), tripling of the genome size in the *S. latifolia* lineage (possibly due to accumulation of TE ^76^) must have tripled the size of the chromosomes, which could have exaggerated peripheral recombination, particularly so on the X chromosome that is the second largest (after the Y) chromosome in *S. latifolia* genome.

Strongly peripheral recombination may have facilitated evolution of recombination suppression between the X and Y chromosome. This conjecture is in line with the growing body of evidence that genome-wide variation in recombination rate, such as reduced recombination in the heterogametic sex (heterochiasmy) ^77–79^, or pericentromeric recombination suppression ^71^ can play a significant role in sex chromosome evolution by facilitating recombination suppression between the X and Y chromosomes. While heterochiasmy is unlikely to be important in *S. latifolia* ^51^, the expansion of pericentromeric recombination suppression may have contributed significantly to evolution of sex chromosomes in this species.

The comparisons of genetic distances for autosomal genes in *S. latifolia* and its relatives would allow us to test whether the expansion of pericentromeric recombination suppression is X-specific, or it occurred on all chromosomes. Unfortunately, with the previous efforts focusing on sex chromosomes, little data is available for autosomes. The genetic distances between 14 autosomal genes in LG1 of *S. latifolia* and *S. otites* are similar in both species (Figure 1d in ^73^), indicating little change in recombination landscape on that autosome over evolutionary time separating these species. Similarly, little difference in recombination distances between three autosomal genes frpericentromeric recombination suppressionom LG9 (E534, E526 and E157) was reported for *S. latifolia* and *S. vulgaris* ^43^. The similarity of genetic distances for analysed autosomal genes in *S. latifolia, S. otites* and *S. vulgaris* suggest that suppression of recombination in the central region of *S. latifolia* X chromosome is probably specific to that chromosome. However, genetic mapping (ideally complemented with physical location) for more genes in several *Silene* species is necessary to reveal the evolution of recombination suppression.

### Extensive pericentromeric recombination suppression on the X chromosome

The rarely recombining Xpr is a large gene poor region spanning ~330Mb in the central part of the *S. latifolia* X chromosome (Figure 2). The lack of recombination in this region explains the clustering of genes near the PAR boundary in the genetic maps published previously ^36,43^. Furthermore, this explains the apparent contradiction between the cytogenetic evidence that placed *SlX1, SlX3, SlCyp* and *DD44X* at the very end of the q-arm on the X chromosome ^52^, while the genetic mapping was consistently placing these genes closer to the middle of the X chromosome map ^36,43^. Our results reveal that these genes are located in the distal region on the q-arm of the X chromosome, which is consistent with the cytogenetic evidence ^52^, while their placement closer to the middle of the genetic maps was caused by the collapse of the physically massive, but rarely recombining Xpr in the genetic maps.

Genes in the qXdr and the Xpr had evolved in very different recombination landscapes before the cessation of recombination between the X and Y chromosomes in these regions. Indeed, the average d*N*/d*S* ratio for the X-linked genes in Xpr is almost two-fold higher compared to the qXdr (Table 2), indicating less effective purifying selection in the former compared to the latter. Partial relaxation of purifying selection in *S. latifolia* X-linked genes was previously reported ^28^, but it was interpreted in the context of lower effective population size of the X-linked compared to autosomal genes rather than reduced recombination on much of the *S. latifolia* X chromosome.

Given the pre-existing recombination suppression in the Xpr, the reduction in the efficacy of selection caused by complete cessation of recombination in the NRY may have been much weaker for the genes in the Xpr compared to the qXdr. This implies that cessation of recombination due to inclusion in the NRY may have had little impact on the already rarely recombining genes in the Xpr. However, given significantly higher d*N*/d*S* for Y-linked compared to X-linked genes in the Xpr (0.591±0.1237 and 0.315±0.0357, respectively; Table 2), inclusion of this region in the NRY resulted in further reduction in the efficacy of selection in Y-linked genes compared to their X-linked gametologs in the Xpr.

Furthermore, the female-bias in gene expression is significantly weaker for the genes in the Xpr compared to the qXdr (Figure 3). While these findings could be interpreted as a result of more recent inclusion of the Xpr in the NRY compared to the qXdr, they are also consistent with the idea that genetic degeneration, caused by complete recombination suppression, affected the qXdr to a greater degree compared to the Xpr that was already rarely recombining. Weak Y-degeneration in the Xpr may, at least partly, account for the previously reported slower rate of Y-degeneration of the *S. latifolia* Y chromosome compared to other studied species ^28,34,59^. However, the slow Y-degeneration was reported for both evolutionary strata in *S. latifolia* ^28^, which cannot be explained by pre-existing recombination suppression as genes in the qXdr undergo frequent recombination on the X chromosome.

## Conclusions

The analyses presented above allow us to reconstruct the likely scenario of sex chromosome evolution in *Silene* and assess its implications for our understanding of evolutionary processes on nascent sex chromosomes. The initial recombination suppression, resulting in formation of NRY, occurred around 11 million years ago ^28^ at the actively recombining end of the proto-sex-chromosomes, corresponding to the qXdr of the *S. latifolia* X chromosome. This event may have been caused by an inversion on the proto-X chromosome, given the order of genes in the qXdr is inverted compared to homologous region in *S. vulgaris* (Figure 4C,D). A few million years later this was followed by NRY expansion via inclusion of the massive Xpr into the male-specific region, possibly driven by evolution of strongly peripheral recombination on the X chromosome. The Xpr region continues to recombine in females, albeit at a very low rate. Recombination suppression in the Xpr may have pre-dated or evolved in concert with NRY expansion. Given the homologous region in *S. vulgaris* is recombining normally (Figure 4D), the latter possibility appears plausible. Either way, reduced recombination in Xpr may have facilitated NRY expansion.

This scenario of sex chromosome evolution in *S. latifolia* deviates from the standard ‘evolutionary strata’ model of step-wise NRY expansion ^67^ when an actively recombining part of the pseudoautosomal region becomes Y-linked and stops recombining, while its homologous region continues to actively recombine on the X chromosome. Consistent with this model, the NRY in *S. latifolia* evolved in an actively recombining region (qXdr), but contrary to that model, the NRY expansion resulted in sex-linkage of a region that may have already been rarely recombining (Xpr), meaning that both X- and Y-linked genes in this region evolve under reduced efficacy of selection. Failing to take into account such pre-existing recombination landscape may lead to misinterpretation in evolutionary genetic analyses of sequence polymorphism and divergence on young sex chromosomes in general.

The scenario described above is compatible with the classic ‘two genes’ model of dioecy and sex chromosome evolution ^33^, with inversion in qXdr preventing recombination between two sex-determining genes, SPF and GSF. The X-linked homolog of the already isolated sex-determining gene (GSF ^72^), along with SlWUS1 that was likely involved in evolution of gynoecium suppression ^72^ are indeed located in qXdr (Figure 2A), though the SPF gene(s) remain to be identified. Our results indicate that X chromosome may play an active role in NRY evolution, with X- (rather than Y-) linked inversions contributing to evolution of recombination suppression between the X and Y chromosomes, which remains to be considered by models of sex chromosome evolution.

## Star Methods

### Resource Availability

#### Lead contact

Further information and requests for resources and reagents should be directed to and will be fulfilled by the lead contact, Dmitry A. Filatov (dmitry.filatov@biology.ox.ac.uk)

#### Materials availability

This study did not generate new unique reagents.

#### Data and code availability

The *S. latifolia* female genome assembly and annotation have been deposited to Genome Warehouse (GWH) database in BIG data Center (https://ngdc.cncb.ac.cn/gwh/) under accession number GWHCBIJ00000000 and BioProject accession PRJCA014197. The PacBio and Hi-C data have been uploaded to NCBI Sequence Read Archive (SRA) database as Bioproject PRJNA952727.

## Experimental Model and Subject Details

### Plant material

*S. latifolia* seedlings for the highly inbred K-line ^80^ were grown in the greenhouse of the Centre for Genomics and Biotechnology at Fujian Agriculture and Forestry University (FAFU) at 22°C under long-day photoperiod (16h of artificial light).

## Method Details

### Genome sequencing and assembly

#### PacBio library construction and sequencing of the female genome

DNA for sequencing was extracted from young leaves of female *S. latifolia* plants. Genomic DNA was sheared and size-selection was carried out using BluePippin system. SMRTbell™ libraries were constructed according to the protocol from PacBio. Subsequently, 104.9 Gb of sequence data were generated with the PacBio Sequel IIe System. PacBio high-quality HiFi reads was generated from subreads by CCS software (v6.4, https://github.com/PacificBiosciences/ccs). HiFi reads were assembled using hifiasm software ^81^ with default parameters, which yielded a 2.64 Gb long contig-level genome assembly (Table S2).

#### Hi-C library construction and sequencing

For Hi-C scaffolding, young leaves of *S. latifolia* female plants from the inbred K-line ^80^ were used to construct the Hi-C libraries by ANOROAD company according to previously published methods ^82^. The Illumina HiSeq X Ten platform was used to sequence the chimeric fragments representing the original cross-linked fragments after constructing the paired-end sequencing libraries. Hi-C reads were uniquely mapped to the contig assemblies and reads within 500 bp regions of HindIII restriction sites were retained for further analysis. A total of 134.8 Gb raw sequencing Hi-C reads were used to assemble the chromosome-level genome using ALLHiC (v0.9.8, https://github.com/tangerzhang/ALLHiC). Hi-C sequencing data were assessed using HiC-Pro ^83^ and the results showed a high proportion of validated reads (59.2%) (Table S4).

#### Chromosome assembly by integration with Hi-C scaffolding and genetic map

The chromosome level genome assembly was integrated with the previously published RNA-seq-based high-density genetic maps ^36,51^, but mostly the sex-specific maps from the recent study ^51^ were used in the analyses as they contained nearly three times more genes than the older sex-average map ^36^. We aligned the sequences of the markers to the *S. latifolia* female contig genome using BLASTN ^84^, retaining BLAST hits with >97% identity longer than 100 bp. As the maps were constructed with transcriptome sequencing ^36,51^, the markers represent actual expressed genes rather than often repetitive non-coding regions, which facilitated finding of these markers in the genome sequence. Due to presence of introns in the genomic sequence of the genetically mapped genes, each gene usually had several adjacent blast hits, corresponding to different exons. The lowest position of the blast hit for each gene was regarded as the genomic position of the particular marker. The correspondence of the positions of markers in the genetic map and genome sequence was checked manually. The contigs were divided into different groups according to the markers of RNA-seq based map, and the group information was combined with the ALLHiC pipeline ^50^. Finally, the contigs were linked into 12 pseudo-chromosomes in the *S. latifolia* female genome.

The integration of the chromosome level assembly with the genetic map revealed minor inconsistencies, which were corrected as follows. The Hi-C reads were aligned to the contigs using the Chromap software (v0.2.4) ^85^. Subsequently the run-assembly-visualizer.sh script from 3D-DNA pipeline (v180922) ^86^ was used to obtain input files for Juicebox Assembly Tools (v2.20.00) ^87^. Then the Juicebox Assembly Tools was used to get the corrected chromosomes.

#### Assessment of accuracy and completeness of the chromosome assembly

The chromatin contact matrix was used to assess the accuracy of chromosome assemblies based on Hi-C by using HiC Explorer (v3.7.2) ^88^. The accuracy and completeness of the assembly was assessed by BUSCO ^89^, and mapping of RNA-seq and genomic Illumina paired end reads. The single base accuracy and the genome coverage rate by the short reads indicated the accuracy of our *S. latifolia* genome assembly (Table S5 and S6).

#### X-chromosome identification

The X chromosome was identified by the presence of X-linked genes from the genetic maps ^36,51^ as well as the X-linked genes identified previously, such as SlX1 ^62^, DD44X ^64^, SlX3 ^60^, SlX4 ^63^, SlssX ^61^, SlCypX ^42^ and the ‘E-genes’ from ^35,43^ (E777, E757, E780, E799, E330 etc).

#### Genome annotation with RNA-seq data

RNA-seq data from the previous study ^36^ were used for *S. latifolia* female genome annotation. Protein-coding genes were annotated based on a previously published approach ^90^. Briefly, RNA-seq transcripts together with homologous proteins were inputted into GETA pipeline (v.1.0, https://github.com/chenlianfu/geta). All parameters were set to the original defaults and the false-positive gene models were filtered by set Pfam database path.

#### Genome filtering and correction

To improve the gene annotation of *S. latifolia* female genome, firstly, the perl script GetaFilter.pl (https://github.com/LengFeng00/biotree.club.git) was used to filter genes that did not meet the standard. The genes were required to meet at least one of the following conditions: FPKM>3, blastp hits with >30% identity and >100 alignment length, containing at least one Pfam domain and Augustus_transcript Support_percentage > 50. Secondly, the IGV-GSAman software (https://gitee.com/CJchen/IGV-sRNA) was used to manually correct and filter out the genes that included TE sequences in coding regions. Finally, the completeness of genome annotation was evaluated by BUSCO.

#### miRNA annotation

For miRNA annotation, the plant miRNAs downloaded from the publicly available database miRBase (most recent access 5 July 2018, http://www.mirbase.org/) were aligned to *S. latifolia* female genome using bowtie ^91^ as described previously ^90^. The mapping results were filtered with PERL script filter_alignments.pl from the miRDP1.3 package ^92^. The miRDeep-P program ^92^, a plant-seecific scoring system explicitly anticipating miRNAs for plants, was used to trim, filter and identification of new miRNAs.

#### Transposable element (TE) annotation

The EDTA v1.9.6 *de novo* annotation tool ^93^ was used to annotate LTR, terminal inverted repeat, and Helitron elements. For EDTA, the following parameters were used in addition to defaults: --step all, --species others, --sensitive 0, --anno 1, and –threads 4. Sequences with multiple paralogs were mapped back to the genome and manually extended to determine the full-length boundary of each TE. A total of 15,560 full-length, representative Copia and Gypsy copies were successfully annotated. The significance of difference in TE abundance between autosomes and the X chromosome was tested with R package ggpubr (version 0.4.0). Significance was tested using the two-sided Mann-Whitney-Wilcoxon test with multiple comparison. P.adjust homl method was used to adjust the P values.

#### Reconstruction of Y-linked genes

To reconstruct Y-linked gametologs for the X-linked genes we followed segregation-based approach described previously ^28,34,36^. In particular, using previously published sequence data from genetic crosses, we called single nucleotide polymorphisms (SNPs) for parents and progeny, including 10 F1 progeny with genome sequence data available ^28^ and 50 F2 progeny with transcriptome data available ^36^. Trimmed RNAseq reads were mapped against the reference genome with BWA mem v0.7.17 ^94^ and sorted with Samtools v1.7 ^95^. Then, SNP calling was done with Samtools mpileup (options: -d 1000 -q 20 -Q 20) and sites filtered with bcftools filter 1.7.

The analysis of SNP segregation in these genetic crosses allowed us to identify Y-linked SNPs that are always inherited from father to sons and never to daughters. The sequence reads containing the Y-SNPs along with their paired reads were separated and assembled into contigs. For this Y-reconstruction procedure we focused on coding regions, while (often repetitive) non-coding regions were excluded from analysis. As described previously ^28,34,36^, this approach allows accurate reconstruction of Y-linked homologs of X-linked genes in *S. latifolia*. The accuracy of the Y-reconstruction was confirmed using the sequences of previously published Y-linked genes (SlY1 ^62^, DD44Y ^64^, SlY3 ^60^, SlY4 ^63^, SlssY ^61^ and SlY7 ^42^).

#### Analysis of gene expression

For gene expression analysis we used previously published RNA-seq data from ^34,36,96^. The analysis of gene expression was conducted with RSEM package ^97^ with default options.

#### Phylogenetic analyses

To analyse substitution rates in sex-linked genes we created three-sequence alignments including X- and Y-linked gametologs as well as a sequence of homologous gene from an outgroup species. As an outgroup we used non-dioecious *Silene uniflora* for which a fragmented genome assembly is already available ^65^. We used CDS sequences of X-linked genes to blast-search the *S. uniflora* genomes. The best-matching homologs were aligned with the X- and Y-linked genes using muscle ^98^. These three-sequence alignments were used for analysis of synonymous and non-synonymous substitution rates in X- and Y-linked genes with codeml from PAML package ^99^. Substitution rates were estimated using branch model ^100^ allowing for separate substitution rate for each branch of the phylogeny.

## Quantification and Statistical Analysis

The Wilcoxon tests were performed using R ggpubr package (version 0.4.0) and the P values were adjusted with p.adjust homl correction in R. Paired t-tests were done in Excel.

**Table:** Key Resource Table

REAGENT or RESOURCE	SOURCE	IDENTIFIER
Deposited Data		
*Silene latifolia* female	This study	GWH: PRJCA014197
genome assembly *Silene latifolia* PacBio	This study	SRA: PRJNA952727
sequences *Silene latifolia* Hi-C data	This study	SRA: PRJNA952727
Experimental Models: Organisms/Strains		
*Silene latifolia* plants	Kazama et al. ^80^	Inbred K-line
Software and Algorithms		
CCS v6.4	NA	https://github.com/PacificBiosciences/ccs
Hifiasm	Cheng et al.^81^	https://github.com/chhylp123/hifiasm
ALLHiC V0.9.13	Zhang et al.^50^	https://github.com/tangerzhang/ALLHiC/wiki
HiC-Pro	Servant et al.^83^	https://github.com/nservant/HiC-Pro
BLASTN	Altschul et al.^84^	https://blast.ncbi.nlm.nih.gov/Blast.cgi
Chromap V0.2.4	Zhang et al.^85^	https://github.com/haowenz/chromap
3D-DNA V180922	Dudchenko et al.^86^	https://github.com/aidenlab/3d-dna
Juicebox Assembly Tools	Durand et al.^87^	https://github.com/aidenlab/Juicebox
HiC Explorer v3.7.2	Wolff et al.^88^	https://hicexplorer.readthedocs.io/en/latest/
BUSCO	Simao et al.^89^	https://busco.ezlab.org/
GETA v1.0	NA	https://github.com/chenlianfu/geta
GetaFilter.pl	NA	https://github.com/LengFeng00/biotree.club.git
IGV-GSAman	NA	https://gitee.com/CJchen/IGV-sRNA
Bowtie	Langmead et al.^91^	https://bowtie-bio.sourceforge.net/manual.shtml
miRDeep-P	Yang et al.^92^	https://github.com/rajewsky-lab/mirdeep2
EDTA v1.9.6	Ou et al.^93^	https://github.com/topics/edta
BWA-MEM	Li and Durbin.^94^	https://github.com/lh3/bwa
Samtools v1.7	Li et al.^95^	https://github.com/samtools/samtools
RSEM	Li et al.^97^	https://github.com/deweylab/RSEM
PAML	Yang et al.^99^	http://abacus.gene.ucl.ac.uk/software/paml.html
branch model	Yang et al.^100^	https://github.com/Bumblebee-Project/Bumblebee/wiki/Branching-Model

## Supplementary Material

Supplementary Materials

## Figures and Tables

**Table 1:** The chromosome lengths and the densities of genes and TEs in *S.latifolia* female genome.

Chromosome	Contigs	Length (bp)	Genes/Mb	P-value*	TE%**	P-value*
ChrX	110	370,597,487	12.1		87.1	
Chr1	188	193,566,110	18.3	7.30E-10	82.8	2.20E-39
Chr2	38	196,073,315	18.4	4.70E-06	83.1	8.90E-26
Chr3	34	165,661,342	18.9	4.60E-11	82.9	4.10E-34
Chr4	52	223,726,948	11.9	0.087	87.3	0.0029
Chr5	51	165,362,148	18.4	8.00E-12	82.7	7.20E-47
Chr6	27	179,322,760	11.2	0.037	87.2	3.00E-05
Chr7	57	193,230,414	14.1	0.16	85.1	0.00017
Chr8	119	172,294,220	15.8	5.20E-06	83.8	5.20E-31
Chr9	55	203,056,505	17.3	0.074	83.5	1.10E-09
Chr10	34	192,831,310	17.6	0.034	83.4	9.10E-14
Chr11	48	196,798,467	14.6	0.02	85.1	3.70E-11

* Significance of difference from the X chromosome (ChrX) was tested using the two-sided Mann-Whitney-Wilcoxon test with P adjust Homl correction. 1 Mb window and 0.2 Mb slide steps were used to calculate the TE abundance and gene density.

** TE% is the % of DNA sequence represented by transposable elements

**Table 2:** Average substitution rates (± standard errors) in the Xpr, qXdr and PAR genes. Non-dioecious *S. uniflora* was used as an outgroup (OG).

	PAR	Xpr	qXdr
#genes	178	760	198
#codons	211,704	839,597	254,442
d*S* X:OG	0.124±0.0068	0.120±0.0042	0.123±0.0070
d*S* X:Y		0.063±0.0018	0.091±0.0042
d*N*/d*S* X		0.315±0.0357	0.267±0.0461
d*N*/d*S* Y		0.591 ±0.1237***	0.408±0.0487***
d*N*/d*S* X:OG	0.221±0.0170	0.285±0.0123	0.233±0.0186

*** Paired t-tests, *P*<0.001, revealing significantly higher d*N*/d*S* for Y-linked compared to X-linked genes in both Xpr and qXdr.
