# Supplementary material for: The origin and evolution of sex chromosomes, revealed by sequencing of the Silene latifolia female genome
Source: Curr Biol. Author manuscript; Available in PMC 2025 Jul 4. (PMC7617815; doi:10.1016/j.cub.2023.05.046)
Supplement: Supplementary Materials [file EMS206174-supplement-Supplementary_Materials.pdf]

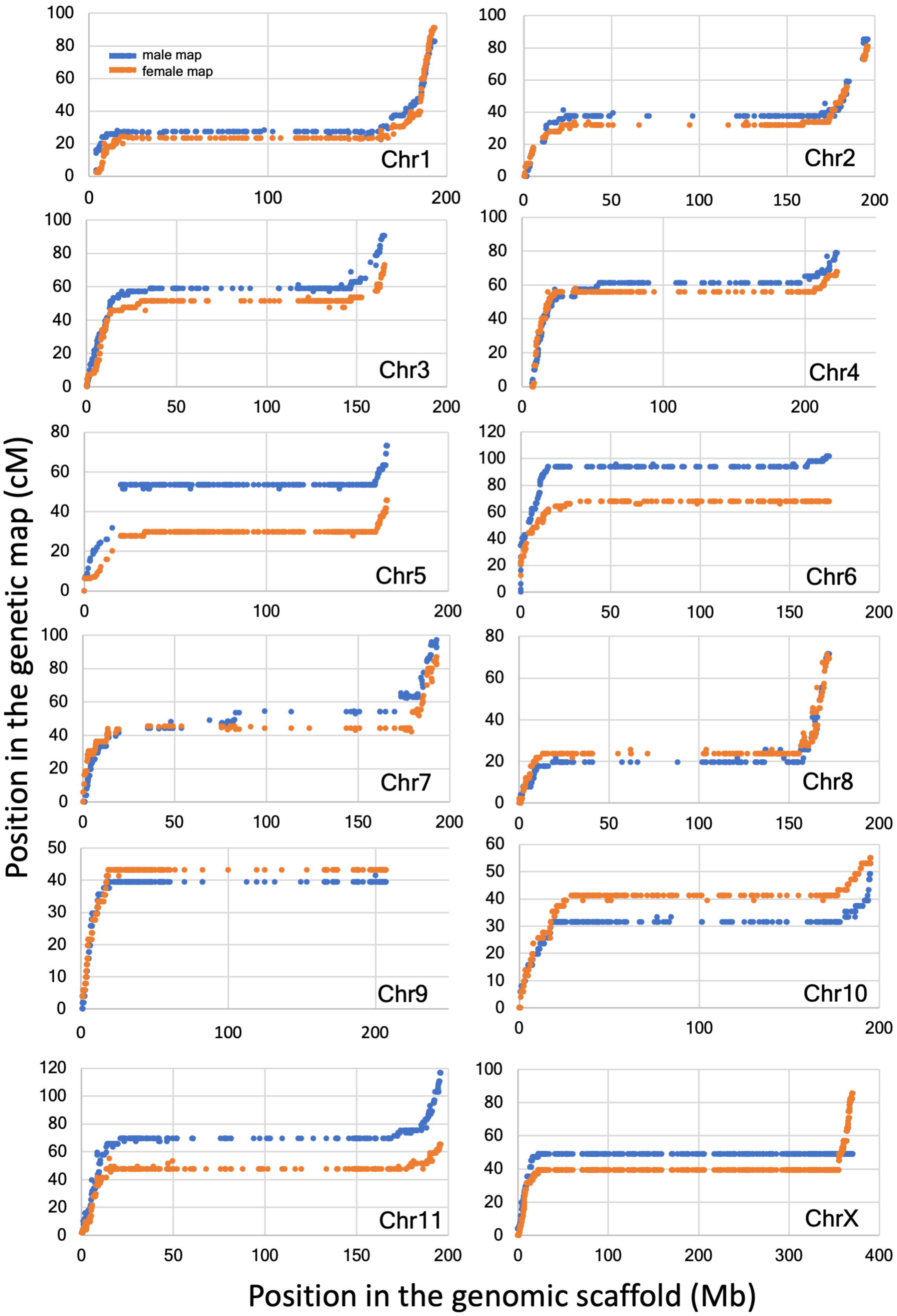

Figure S1. **Marey maps relating the position of genes in the genetic map to physical positions for *S. latifolia* chromosomes. Related to Figure 2**. Female map is in orange, male map is in blue.

	PacBio HiFi	Illumina-HiSeq	Illumina-MiSeq
Total number of reads	6,627,172	219,465,897	41,281,143
Total number of bases (Gb)	104.94	21.81	12.43
Mean reads length (bp)	15,835	100	300
N50 (bp)	15,753	100	300
Coverage	40	8	5

Table S1. Sequencing information of *S. latifolia* female genome. Related to Figure 1.

Items	Statistics of S. *latifolia* assembly
Total assembled size (bp)	2,638,531,022
Number of contigs	5,426
N90 (bp)	1,342,000
N80 (bp)	6,485,999
N70 (bp)	11,834,000
N60 (bp)	17,744,000
N50 (bp)	23,141,000
Average length (bp)	486,275
Maximum length (bp)	80,397,000
Number of anchored contigs	4,613
Anchor rate (%)	92.95%
Length of chromosome level assembly	2,452,521,026
Annotated protein-coding genes	37,796
Annotated miRNAs	96

Table S2. Assembly of *S. latifolia* female genome. Related to Figure 1.

Description	Number	%
Complete BUSCOs(C)	1582	98.0
Complete and single-copy BUSCOs(S)	1461	90.5
Complete and duplicated BUSCOs(D)	121	7.5
Fragmented BUSCOs(F)	14	0.9
Missing BUSCOs(M)	18	1.1
Total BUSCO groups searched	1614	100

Table S3. BUSCO analysis of *S. latifolia* female genome completeness. Related to Figure 1.

Statistics of read mapping	
Clean paired-end reads	826,594,344
Unmapped paired-end reads	5,516,535
Unmapped paired-end reads rate (%)	0.667
Paired-end reads with singleton	88,839,941
Paired-end reads with singleton rate (%)	10.748
Multi mapped paired-end reads	0
Multi mapped rate (%)	0
Unique mapped paired-end reads	280,338,931
Unique mapped rate (%)	33.92
Statistics of valid reads	
Dangling end paired-end reads	28,710,601
Dangling end rate (%)	10.24
Self circle paired-end reads	679,936
Self circle rate (%)	0.24
Dumped paired-end reads	14,300
Dumped rate (%)	0.005
Interaction paired-end reads	212,602,755
Interaction rate (%)	75.84
Lib validpaired-end reads	165,855,542
Lib valid rate (%)	59.16
Lib dup rate (%)	78.01

Table S4. Statistics of Hi-C sequencing and mapping of *S. latifolia* female genome. Related to Figure 1.

	Number	Total Length (bp)	Accuracy (%)	Bases covered by assembly (%)	Sequences covered by assembly (%)	With >90% sequence in same chromosome		With >50% sequence in same chromosome	
						Number	Percent	Number	Percent
Assembled transcripts	44,537	51,165,399	98.41%	99.90%	99.99%	44,448	99.80%	44,530	99.98%

Table S5. Assessment of *S. latifolia* female genome based on RNA-seq assembled transcripts. Related to Figure 1.

Items	*S. latifolia* genome
Number of reads	18,985,526
Data size (Gb)	178.35
Mapped bases (Gb)	49.32
Mapping rate (%)	99.08%
Genome length (Mb)	2.31
Mean depth	19.93
Coverage rate (%)	93.97%
Regions with low coverage (<5 reads)	251,790,000
Percentage with low coverage (<5 reads)	10.9%
Number of homozygous variants	3,729,152
Percentage of homozygous variants	0.16%

Table S6. Assessment of genome consistency based on genomic Illumina reads. Related to Figure 1.

Description	Number	%
Complete BUSCOs(C)	1,563	96.9
Complete and single-copy BUSCOs(S)	1,431	88.7
Complete and duplicated BUSCOs(D)	132	8.2
Fragmented BUSCOs(F)	23	1.4
Missing BUSCOs(M)	28	1.7
Total BUSCO groups searched	1,614	100

Table S7. BUSCO analysis of gene completeness. Related to Figure 1.

	Number	Length (bp)	% of repeats	% of genome
**Total repeats**	2,543,673	2,184,279,006	100	82.78
**LTR**	1,791,894	1,892,656,091	86.65	71.72
Copia	294,426	336,782,852	15.42	12.76
Gypsy	658,104	946,294,079	43.32	35.86
unknown	839,364	609,579,160	27.91	23.10
**TIR**	491,286	200,990,831	9.20	7.62
CACTA	109,195	58,077,166	2.66	2.20
Mutator	179,490	81,817,291	3.75	3.10
PIF_Harbinger	22,826	7,183,645	0.33	0.27
Tc1_Mariner	114,873	32,073,778	1.47	1.22
hAT	64,902	21,838,951	1.00	0.83
**nonTIR**	259,992	90,549,730	4.15	3.43
helitron	259,992	90,549,730	4.15	3.43

Table S8. Transposable elements in *S. latifolia* female genome. Related to Figure 1.

Region	Compared	P-value	p.adj	p.signif
Genes:				
PAR	Xpr	6.49E-73	1.90E-72	****
qXdr	PAR	0.69304886	0.69	ns
Xpr	qXdr	5.38E-38	1.10E-37	****
TEs:				
PAR	Xpr	3.38E-75	1.00E-74	****
qXdr	PAR	0.926265516	0.93	ns
Xpr	qXdr	4.25E-38	8.50E-38	****

Table S9. The comparisons of gene and transposable element (TE) density in three regions of *S. latifolia* X chromosome. Related to Figure 1.

* Significance was tested using the two-sided Mann-Whitney-Wilcoxon test. P adjust homl method was used to adjust p values. 1 Mb window and 0.2 Mb slide steps were used to calculate the gene density.
